# Multi-Faceted Notch in Allergic Airway Inflammation

**DOI:** 10.3390/ijms20143508

**Published:** 2019-07-17

**Authors:** Miao-Tzu Huang, Chiao-Juno Chiu, Bor-Luen Chiang

**Affiliations:** 1 Department of Medical Research, National Taiwan University Hospital, Taipei 10048, Taiwan; 2 Department of Pediatrics, National Taiwan University Hospital, Taipei 10048, Taiwan; 3 Graduate Institute of Clinical Medicine, School of Medicine, National Taiwan University, Taipei 10048, Taiwan

**Keywords:** notch, jagged, delta-like, allergic airway inflammation, airway remodeling, angiogenesis, short-chain fatty acids, regulatory T cells

## Abstract

Notch is an evolutionarily conserved signaling family which iteratively exerts pleiotropic functions in cell fate decisions and various physiological processes, not only during embryonic development but also throughout adult life. In the context of the respiratory system, Notch has been shown to regulate ciliated versus secretory lineage differentiation of epithelial progenitor cells and coordinate morphogenesis of the developing lung. Reminiscent of its role in development, the Notch signaling pathway also plays a role in repair of lung injuries by regulation of stem cell activity, cell differentiation, cell proliferation and apoptosis. In addition to functions in embryonic development, cell and tissue renewal and various physiological processes, including glucose and lipid metabolism, Notch signaling has been demonstrated to regulate differentiation of literally almost all T-cell subsets, and impact on elicitation of inflammatory response and its outcome. We have investigated the role of Notch in allergic airway inflammation in both acute and chronic settings. In this mini-review, we will summarize our own work and recent advances on the role of Notch signaling in allergic airway inflammation, and discuss potential applications of the Notch signaling family in therapy for allergic airway diseases.

## 1. Introduction

### 1.1. Allergic Airway Diseases

Allergic asthma has long been considered as a genetically predisposed Th2 immune-mediated inflammatory disease and may causally be attributed to impaired regulatory T cell (Treg) immune regulation [1,2,3,4,5,6].

However, the phenotypic heterogeneity of asthma and unfavorable results of Th2-directed therapies in a group of individuals have led to the appreciation of non-Th2 immune reaction in pathogenesis of allergic airway inflammation and clinical re-categorization of Th2-high and Th2-low endotypes of asthma [7]. Airway inflammation in asthma is elicited when the airway epithelium of a previously sensitized individual encounters seemingly harmless environmental factors, such as pollen, house dust mites, and cat dander. Allergic airway inflammation is characterized by IgE-dependent activation of the mast cells and infiltration of eosinophils and activated Th2 lymphocytes into the airway mucosa. Mast cells can be directly activated by allergens through cross-linking of the surface-bound IgE. This causes rapid release of preformed mediators from the mast cells, including histamine, leukotrienes and prostaglandins, resulting in bronchoconstriction and airway mucosa edema due to vasodilatation and plasma exudation, which culminate in symptoms of asthma [8]. In parallel, allergen exposure induces secretion of IL-33, IL-25, and TSLP by the airway epithelia, which activate the mast cells, innate lymphoid cells (ILC)-2 and dendritic cells to propagate the Th2-skewed responses, causing airway eosinophilia, mucus production, and hyperreactivity of the airways [9,10]. The resulting pathology due to recurrent allergic inflammation of the airways includes goblet cell metaplasia, subepithelial matrix deposition, airway smooth muscle hypertrophy, and hyperplasia, which cumulatively lead to irreversible impairment of lung functions [7,9].

Avoidance of allergens that trigger asthma and long-term medicinal control remain the mainstay in prevention of asthma attacks. Despite advances in understanding of the mechanisms underlying the pathogenesis of asthma, inhaled corticosteroids (ICS) are by far the most effective treatment modality for asthma due to their capacity to unequivocally suppress the characteristic inflammation of the asthmatic airways. 

Recently, new therapies specifically targeting IgE and the canonical Th2 cytokines IL-5, IL-4 and IL-13 have been developed based on knowledge of the molecular mechanisms mediating asthmatic airway inflammation. These targeted immunotherapy, in particular anti-IgE and anti-IL5 antibodies, have been shown to reduce asthma severity and frequency of acute exacerbation and achieve steroid-sparing effect in patients with Th2-skewed asthma endotype [11,12,13,14]. In addition, the mechanisms underlying these Th2-directed immunotherapies have been shown to go beyond mere blockage of the targeted molecules. Regulatory mechanisms including IgG4 antibodies, which are capable of inhibiting the IgE-dependent inflammatory cascade, deletion, or anergy of antigen-specific T effector cells, induction of Tregs and Th1 immune deviation have been demonstrated following immunotherapy and are believed to contribute to the therapeutic effects of immunotherapy. However, successful trials of targeted therapies in asthmatic patients without evidence of Th2 inflammation are still lacking [15,16]. Investigations into the molecular mechanisms of the non-Th2 asthma endotypes or pathways upstream of the Th-skewing stage are therefore mandatory.

### 1.2. Notch Signaling Pathway

Notch is an evolutionarily conserved signaling family, regulating a diverse array of cellular behaviors and physiologic events in neuronal, cardiovascular, respiratory, hematologic, immune and endocrine systems from the embryonic stage through the entire lifespan. Aberrant Notch signaling leads to abnormal development, dysfunctional biologic processes, and malignant transformation [17,18,19,20,21,22,23,24,25,26,27]. The Notch signaling pathway has been an interesting target for pharmacological intervention owing to its pleiotropic and crucial roles in human diseases. 

The mammalian Notch family includes four Notch receptors, Notch-1, -2, -3, and -4 and five Notch ligands, Jagged-1, and -2, and Delta-like ligand (DLL)-1, -3, and -4; the ligands are homologues of Delta, Serrate, and Lag2 (DSL) in invertebrates [28,29]. Both Notch receptors and ligands are cell transmembrane proteins, hence restricting Notch signaling to the neighboring cells or between cells in close proximity. In addition, individual cells may simultaneously express both Notch ligand and receptor. Therefore, the Notch pathway is unique in that it mediates juxtacrine and lateral cellular signaling. Notch receptors are single-pass transmembrane proteins composed of extracellular (NECD), transmembrane (TM), and intracellular (NICD) domains. Intracellular transduction of the Notch signal is transmitted without secondary messengers, rather through the NICD. Upon Notch ligand binding, the NECD is cleaved away from the TM-NICD domain by TACE (TNF-α ADAM metalloprotease converting enzyme). The NICD is released from the TM domain by γ-secretase intramembrane proteolytic cleavage and translocates into the nucleus, where it displaces transcriptional repressors and associates with PBPJκ/CSL (CBF1/Su(H)/ Lag-1) and the co-activator Mastermind-like family (MAML) to form the transcriptional activation complex of the target genes. Binding of Notch receptors by DLL and Jagged (or DSL), and transcriptional activation involving PBPJκ/CSL is considered the canonical Notch pathway, activating the downstream target genes such as Myc, p21, and the HES family [30,31,32,33].

However, given the limited numbers of the Notch ligands and the common downstream canonical signaling cascade irrespective of Notch receptor-ligand pairs, it is difficult to explain the pleiotropic functions as exerted through the canonical Notch pathway. Several theories have been proposed for this issue. It is now believed that non-canonical ligands that include a group of integral and glycosylphosphatidylinositol (GPI)-linked membrane as well as secreted proteins are, at least in part, responsible for the diverse functions attributed to Notch signaling [34,35]. In addition, there are studies showing a context-dependent effect wherein Notch signaling receives and integrates additional signals derived from the surrounding environment, such as those from other membrane receptors and cytokines secreted by activated parenchymal or immune cells [27,36,37]. In this case, the Notch pathway is described as a signaling hub that collects messages arising from the signaling cell per se, but also from the environment, either cellular or secreted factors. Alternatively, the fact that engagement of distinct Notch ligands induces signals that differentially regulate T-cell differentiation and activation has led to the theory that ligand-specific Notch signaling can be generated by the characteristic expression patterns of Notch ligands under various inflammatory conditions [38,39]. In addition, Fringe-mediated glycosylation of the Notch receptors in the Golgi has been shown to affect the signaling competence of unique Notch receptor-ligand pairs [40,41]. In this circumstance, the glycosylation pattern of Notch receptors endows preferential ligation by particular ligands rather than others. This theory undoubtedly lays the basis for targeted therapeutic approaches exploiting the ligand-specific Notch signaling pathway. Nonetheless, different experimental systems and approaches, such as deletion/inactivation, versus overexpression/ligation, versus pharmacological inhibition, may also cause different Notch signaling results. 

In this mini-review, we will discuss the role of Notch signaling in the pathogenesis of allergic airway inflammation in both acute asthma attacks and under chronic settings with airway remodeling. Through this review we will share our view on the use of the Notch pathway as a potential target in the treatment of allergic airway diseases. 

### 1.3. Notch in Lung Development

In the context of the respiratory system, it has been demonstrated that Notch signaling plays an essential role during lung development. In the developing lung, the pluripotent epithelial progenitors residing in the terminal buds of elongating airways give rise to lineage-restricted progenitors of the conducting airways via processes coordinated by Notch. The epithelial early proximal-distal fate decisions, secretory Clara vs. ciliated cell committed differentiation, establishment of pulmonary neuroendocrine cell populations, as well as airway branching, alveologenesis and pulmonary vascular development, are all under regulation by Notch [42]. Given the function of Notch signaling in stem cell differentiation, cell proliferation and apoptosis, it is conceivable that Notch signaling is also involved in the response and repair of lung injuries, such as in chronic obstructive pulmonary disease, lung fibrosis, asthma and lung cancers [26].

## 2. Notch Signaling in Acute Allergic Airway Inflammation

### 2.1. Notch in Th Subset Differentiation and Immune Responses 

Among the pleiotropic functions of Notch in cell development and tissue homeostasis, Notch has been shown to shape the establishment of peripheral immune system and immune responses by generating instructive signals for T helper (Th)-cell subset differentiation, such as Th1, Th2, Th9, Th17 as well as Tregs. The mechanisms underlying Notch regulation of Th subset differentiation have been attributed to the induction of lineage-specific transcription factors and the signature products, such as cytokines produced by distinct T-cell lineages [37,43]. IL-10 is an anti-inflammatory cytokine and expression of IL-10 by Th1 cells is an essential self-regulatory pathway to limit the inflammatory responses. In addition to regulating the Th-subset development mentioned above, Notch signaling was also found to induce Th1-cell production of a large amount of IL-10. These Th1 cells subsequently lost their inflammatory capacity and were instead immunosuppressive in an IL-10-dependent manner. IL-10 production can be elicited by all four mammalian Notch receptors, but was selectively induced by the DLL family. Studies have shown that dendritic cells (DCs) upregulated DLL4 expression upon stimulation with various TLR ligands and the acquisition of DLL4 conferred DCs the ability to induce IL-10 production by Th1 cells [44]. Later studies demonstrated that plasmacytoid DCs also instructed T-cell production of IL-10 through the DLL4-Notch signaling pathway [45].

Previous studies have demonstrated that Notch induces differential T-cell activation and differentiation programs via distinct Notch ligand engagement. In studies using DLL vs. Jagged gain-of-function approaches, DLL engagement preferentially induced Th1 differentiation, whilst Jagged generated signals that favor Th2 [37,38]. In line with these findings, by using infectious disease animal models, it has been shown that DLL4-derived Notch signaling induced activation of Th1 and Th17, which exerted anti-viral and anti-mycobacterial immune responses, respectively [46,47]. These results are indicative of ligand-specific effects of the Notch pathway in induction and shaping of immune responses, not only in vitro but also with impacts on disease pathogenesis and outcome. γ-secretase inhibitor (GSI) is a general inhibitor of the Notch signaling pathway, which acts by blockage of the Notch intramembrane cleavage, and hence prevents the subsequent release of NICD signaling molecule [48,49]. Although experimental inhibition of Notch signaling by using GSI has shown feasibility in potential applications of Notch in therapies for cancers and immunologic diseases [48,50,51], non-specific inhibition of Notch may lead to detrimental side effects owing to the plethoric biologic events that Notch participates in, especially those associated with cell proliferation and renewal. In addition to catalyzing NICD release, γ-secretase is known to cleave over 100 type-I membrane proteins. It is conceivable that substrate-selectivity may affect the druggability of γ-secretase and GSI. Considering the ligand-specific effects of Notch signaling and the toxicity related to non-selective inhibition of GSI, investigation into ligand-specific Notch targeting is mandatory.

In the context of allergic airway inflammation, the involvement of Notch has been demonstrated in various preclinical studies by using GSI (Table 1). In one of these studies, administration of GSI alleviated asthma phenotypes, including mitigated eosinophilic airway inflammation, goblet cell metaplasia, and airway hyperreactivity, as well as decreased Th2 cytokine secretion and allergen-specific IgE production [51]. In an alternative approach, GSI-treated CD4^+^ and CD8^+^ T cells were unable to convey Notch-induced airway inflammation and hyperresponsiveness in allergen-sensitized mice [52,53], indicating that Notch regulates the pathogenesis of allergic airway inflammation via signaling through T cells. In humans, CD4^+^ T cells in peripheral blood taken from patients with asthma express higher levels of Notch than the healthy controls [54]. In addition, Notch signaling has been shown to be essential for eosinophil chemotaxis and transendothelial migration, implying a role of Notch in eosinophil recruitment into the tissues during allergic inflammation [55,56]. Gene network analysis of the public transcriptomics datasets for genes and pathways involved in epithelial barrier dysfunction of asthma has uncovered Notch2 as one such gene. The association of insufficient Notch signaling and barrier dysfunction in asthma as discovered with network transcriptomics analysis was verified in human airway epithelial brushings and primary asthmatic epithelial cells. [57]. Furthermore, in a genomewide association study (GWAS), Notch was found to be protective of developing severe airway hyperresponsiveness in adult asthma [58]. These findings depict the pleiotropic nature of Notch signaling in allergic airway inflammation where Notch regulates the development of asthma not only through both the innate and adaptive immunologic compartments, but also by regulation of airway epithelial differentiation and function.

### 2.2. Notch Ligand-Specific Allergic Immune Responses

Involvement of the Notch pathway in allergic airway inflammation has been further dissected by using ligand-specific over-expression, knockouts, recombinant Notch-ligand proteins and blocking antibodies. These studies have advanced our understanding of the specific contribution of individual Notch ligands, especially DLL versus Jagged, in allergic inflammation (Table 1) [59].

Collectively, previous studies have concluded a role for Jagged, and mainly Jagged1, in promoting Th2-cell differentiation and Th2-skewed inflammation [38,52,60,61,62]. In addition, Jagged1-mediated Notch signaling regulates secretory cell differentiation of the human airway epithelium [63]. On the other hand, Notch signals derived from DLL ligation have been shown to antagonize Jagged-induced Th2 responses [64,65,66,67]. In this aspect, studies have shown that DLL4 mitigated allergic airway inflammation by induction of Th2-cell apoptosis [67]. Th2-mediated allergic airway responsiveness was reverted towards a Th1 phenotype after administration of exogenous DLL1-Fc fusion protein. In line with the Th1/Th2 counteractive effect between DLL and Jagged, the protective Th1 anti-viral response generated during respiratory syncytial virus infection was complicated by severe Th2-mediated pulmonary inflammation when the infection occurred in the absence of DLL4 signals [68].

However, in addition to the antagonistic effect of DLL4 and Jagged1 in generating Th1 versus Th2 inflammatory response, the findings that inhibition of DLL4 signals led to aggravated Th2 inflammation and enhanced disease severity have prompted further investigation of alternative effects and mechanisms of DLL4-elicited signals [46,67,69].

In an in vivo murine model, high-dose intranasal peptide delivery induced the emergence of DLL1-expressing T cells, which inhibited activation of neighboring T cells and thereby established immune tolerance by direct cell-cell contact and signaling through DLL1–Notch1 [70]. Despite the early date of the study, it provided the very first clue that Notch signaling plays a role in Treg differentiation and immune regulation. By using the murine allergic asthma model, we have shown that blockage of Jagged1 signaling mitigated Th2-dominated airway inflammation, whilst inhibition of DLL4 signaling aggravated the asthma phenotypes. We further demonstrated that DLL4 induced the immunosuppressive Treg population, whereas DLL4 signaling blockage hampered Treg expansion. Hence, DLL4 not only counteracts Jagged-induced Th2 response, it additionally elicits endogenous regulation of inflammation through induction of Tregs, which suppressed propagation of the inflammatory cascade and contributed to resolution of the allergic inflammation [69]. It has come to our attention that inflammatory responses adopt the “Yin and Yang” modality to keep the inflammation in check. Overexuberant and uncontrolled inflammation leads to autoimmunity and organ damage. It is therefore plausible that Jagged initiates the Th2 inflammation in the lung, whilst simultaneously DLL4 signaling generates the Treg homeostatic self-regulatory pathway. In line with our findings, similar results were also obtained during respiratory syncytial virus infection. In this model, inhibition of DLL4, as was highly expressed by DCs of the lung and draining lymph nodes, decreased abundance of CD62L^hi^CD44^lo^Foxp3^+^ Tregs, resulting in exacerbated mucus secretion, increased Th2 cytokine IL-5 and IL-13 production, as well as concomitant expansion of the Th17 cells. In contrast, DLL4 signal induced differentiation of Tregs with enhanced suppressive function and endowed resistance to Th17 skewing [46].

DLL4 signaling has been shown to also induce Th17 differentiation in the presence of skewing cytokines, IL-6 and TGF-β. These studies demonstrated that DLL4 up-regulated Rorc expression in T cells and that Rorc and Il17 genes are both direct Notch transcriptional targets. In the absence of DLL4-Notch signals, IL-17 production was significantly inhibited even under Th17-skewing conditions [47,71]. In parallel, similar findings were also observed in γδ-T cells, wherein DLL4 supported the differentiation of IL-17-producing γδ-T cells [72]. For the dual role of DLL4-Notch signaling in differentiating Th17 and Tregs, studies have shown that DCs act as a crucial balancer of Th17 vs. Treg differentiation through direct Rbpj transcriptional activation of the Aldh1A2, the gene encoding aldehyde dehydrogenase 1A2, also known as retinaldehyde dehydrogenase 2 (RALDH2). The product of Aldh1A2 is an enzyme that catalyzes retinoic acid synthesis from retinaldehyde. Therefore, Rbpj-deficient DCs expressed low level of Aldh1A2 and exhibited reduced ability to induce Treg differentiation by TGF-β. On the other hand, Aldh1A2 overexpression overcame the Th17-differentiating ability of Rbpj-deficient DCs [73].

Recent studies have uncovered Th17 as the non-Th2 new player in the pathogenesis of allergic airway inflammation, causing neutrophilic inflammation of the airways and poor steroid treatment response [9,74,75,76]. It is conceivable that Notch may participate in allergic airway inflammation via the Notch-Th17 axis. In this aspect, pharmacological inhibition of Notch by using γ-secretase inhibitors and Notch-DLL4 signaling blockage by antibodies suppressed Th17 development and decreased asthma severity [77,78]. In addition, dysregulated Treg vs. Th17, accompanied by elevated Notch expression, were found in asthmatic patients [79]. Therefore, in addition to regulating Th1 vs. Th2 development, Notch also orchestrates the development of allergic airway inflammation through balancing Treg versus Th17, suggesting Notch as a potential therapeutic target for both Th2 and non-Th2 asthma endotypes.

### 2.3. Contradictory Findings

**Figure 1:**
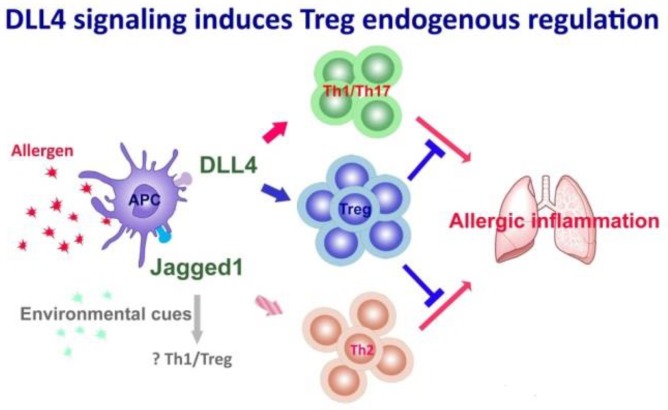
Delta-like ligand 4 (DLL4) and Jagged1 exert different effects on Th subsets differentiation. Whilst Jagged1 promotes Th2-mediated allergic inflammation in the lung, DLL4 signaling induces Tregs as the endogenous regulatory pathway, contributing to resolution of the allergic inflammation.

With regard to the role of DLL4-Notch signaling in Treg differentiation, studies using different disease models have also shown contradictory results. In experimental autoimmune encephalitis (EAE), type 1 diabetes and GVHD disease models, blockage of DLL4 reduced disease severity through induction of Tregs [82,83,84]. In addition, Jagged1, by engaging CD46, induced Th1-cell differentiation and their subsequent conversion into IL-10-producing regulatory T cells [85,86]. Other studies supporting a role for Jagged1 in Treg differentiation have demonstrated that T cells upregulated production of TGF-β, whilst at the same time reduced expression of IL-2, IFN-γ, and IL-5, after Jagged1 ligation [87]. It is not yet fully understood what leads to these discrepant results. Notch has been shown to act as a signaling hub receiving signals originated from cell surface molecules or the adjacent milieu, that is, the environmental cues. It is conceivable that the overall Notch signaling outcome is modulated in a context-dependent manner [27,34,35,37,39,88]. Therefore, the nature of the inflammatory response with its characteristic Th cytokine profile per se, as well as the activation of other molecules during the inflammatory process which subsequently interact with Notch will very likely regulate differential Notch signaling results (Figure 1).

## 3. The Role of Notch Signaling in Chronic Allergic Airway Inflammation

### 3.1. Airway Remodeling and Angiogenesis in Chronic Asthma

In asthma, repeated airway inflammation leads to airway remodeling [89,90,91], which is now believed to persist beyond resolution of the allergic inflammation [92,93]. Airway remodeling is a causal factor for persistent airway hyperresponsiveness (AHR) in asthmatic patients and is responsible for the irreversible lung function impairment [89,90,91,94,95], which is at best only partially relieved by corticosteroids in both experimental and clinical settings.

The structural alterations in asthmatic airways are attributed to various functional effects of the eosinophils [96], mast cells [97], macrophages and Th2 cytokines, IL-4, IL-5, IL-13 and TGF-β [94,98]. The pathologic findings include airway epithelial hyperplasia, goblet cell metaplasia, airway smooth muscle hypertrophy and deposition of extracellular matrix (ECM). In addition to remodeling of the epithelial-ECM components, increased subepithelial vascularity, vasodilatation and microvascular leakage are also observed [99,100,101]. Deposition of angiogenic factors VEGF, bFGF and angiogenin have been found in the airway submucosa [101,102,103]. Among these angiogenic factors, VEGF is able to propagate the Th2 inflammation and in a feed-forward manner, aggravates airway remodeling [104].

### 3.2. Notch Signaling Family and Angiogenesis

Among its pleiotropic functions, the Notch signaling pathway is also involved in multiple aspects of angiogenesis, including endothelial cell (EC) proliferation and migration, smooth muscle differentiation and vascular patterning and stabilization of the vascular networks [105]. Regulation of angiogenesis by Notch requires spatial and temporal coordination, since constitutive activation or inhibition of Notch signaling leads to abnormal vascular formation [105,106,107].

Previous studies have shown that VEGF and bFGF enhanced Notch1 and DLL4 expression of the EC [108,109]. The up-regulated DLL4 in turn inhibited VEGF signaling and function by down-regulating EC expression of VEGF-R2 [110]. DLL4-elicited negative-feedback regulation restrains vascular sprouting and branching. Hence, DLL4 signaling appears to exert a negative regulatory role in angiogenesis, preventing excessive vessel formation to maintain a functional vascular network. Therefore, the DLL4 negative regulatory effect can be beneficial in the inflammation-remodeling angiogenesis reciprocal reinforcing cascade, which is maintained at least in part by VEGF [111,112]. In parallel, the negative regulatory role of DLL4-Notch signaling in angiogenesis has been demonstrated in studies by using genetic deletion/overexpression, recombinant proteins, or blocking Abs [110,113,114,115]. In these studies, DLL4-Notch signaling blockage caused aberrant endothelial proliferation, which culminated in impaired maturation and poor perfusion of the vessels [116,117,118].

**Figure 2:**
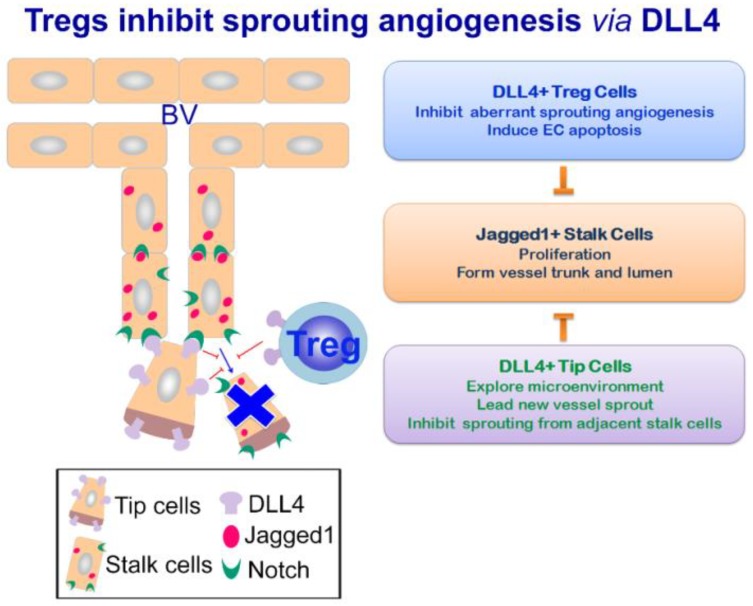
Tregs inhibit sprouting angiogenesis of the airway remodeling by DLL4 signaling. During chronic airway inflammation, DLL4 expression by Tregs is able to ameliorate aberrant angiogenesis of the remodeling airways by inhibiting vessel sprouting. Decreased non-functional angiogenesis within the airway mucosa leads to improved lung function.

It is well-known that Jagged1 and DLL4 act in an antagonistic manner in many biologic events, and this is also the case in angiogenesis. In growing new vessels, DLL4 is highly expressed by the tip cells at the leading front of the vascular sprouts. Tip cells inhibit endothelial sprouting from neighboring stalk cells by DLL4 engagement of the Notch receptors on the stalk cells, which induces signals downregulating VEGF-R2 expression on the stalk cells [114,119]. Therefore, DLL4-Notch engagement delivers angiogenic “off” signals, rendering the EC unresponsive to angiogenic cues such as VEGF and pro-angiogenic inflammatory cytokines, which would otherwise cause aberrant angiogenesis and generate non-functional vasculature. Therefore, DLL4-Notch signaling maintains the vascular network in a mature and functional state [109,110,120]. In contrast to the anti-angiogenic effect of DLL4, Jagged1 is a potent pro-angiogenic regulator abundantly expressed in the stalk cells of the vessel sprouts. In the stalk cells, Jagged1 antagonizes DLL4-Notch signaling and maintains an active angiogenic phenotype of the stalk cells, thereby promoting angiogenesis [121]. It is plausible that Notch ligands DLL4 and Jagged1, through their distinct spatial expression patterns and opposing angiogenic functions, maintain equilibrium during angiogenesis [121,122] (Figure 2).

### 3.3. Tregs Ameliorate Remodeling Angiogenesis by DLL4-Notch Signaling

The Notch signaling pathway is known to regulate peripheral T-cell differentiation and hence orchestrate progression of the immune reaction [37,43,59,123]; however, the role of the Notch pathway in chronic allergic inflammation in the lung is not clear.

Tregs have been thoroughly investigated in the past two decades due to their unique immune suppressive effects. Previous studies unequivocally demonstrate a crucial role for Tregs in mitigating the severity of allergic airway inflammation and preventing acute exacerbation of allergic asthma. Nonetheless, in addition to exerting immune regulatory effects under acute aspect of the allergic airway inflammation, we have previously found that Tregs ameliorated severity of chronic allergic asthma with established airway remodeling and impaired lung function. By therapeutic transfer of Tregs at chronic stage of the disease, we found that Tregs improved lung function by selectively modulating the pulmonary vascularity of the remodeled airways [124].

We found Tregs expressed significantly higher levels of Notch ligand DLL4 compared to their T-effector counterparts. Due to the negative regulatory role of DLL4-Notch signaling in sprouting angiogenesis, Tregs may regulate remodeling angiogenesis by engaging the proliferating EC with DLL4 to deliver angiogenic “off” signals. Aberrant remodeling angiogenesis leads to impaired lung function. In this aspect, Tregs-elicited DLL4-Notch signals would be beneficial in preventing non-functional poor-perfused angiogenesis and ameliorating perfusion/ventilation abnormality as often seen in chronic asthma. We confirmed the anti-angiogenic effects of Tregs by using both in vitro and in vivo angiogenesis assays. Decreased EC angiogenesis was found in the presence of Tregs. In addition, decreased circulating endothelial progenitors as well as pro-angiogenic cells were also observed after Treg transfer. The anti-angiogenic function of Tregs was abrogated by treating Tregs with Notch-Fc proteins or an anti-DLL4 blocking Ab. In line with these results, Tregs pretreated with the DLL4 blocking Ab failed to improve the asthma phenotype and lung function of the recipient mice after adoptive transfer (Figure 2).

Although a prominent role of Tregs in regulating allergic airway inflammation is well-defined, regulation of EC angiogenesis by Tregs has not been reported before our studies. Collectively, we have identified a novel pathway for Tregs in chronic allergic airway disease, whereby Tregs cross talk with EC via DLL4 signaling, inhibit remodeling angiogenesis and improve pathology of the remodeled airways, which ultimately leads to improved lung function. The findings that Tregs alleviate inflammation-induced angiogenesis have important implications for clinical application of Tregs, especially for chronic immunologic diseases such as rheumatoid arthritis and asthma that are often accompanied by pathologic angiogenesis [112,125]. Tregs in this condition exert a dual role that not only tunes down the inflammation but also targets the detrimental inflammatory consequence of pathological angiogenesis.

The unique function of DLL4-Notch signaling in angiogenesis has uncovered a novel therapeutic target for various diseases where angiogenesis plays a role in perpetuating the disease pathogenesis. Indeed, in the past decade, clinical trials have been undertaken by utilizing anti-DLL4 Ab alone or in combination with VEGR blockade for better cancer treatment outcomes [126,127,128,129]. In the aspect of allergic airway disease, we are awaiting this novel and promising DLL4 angiogenesis pathway being applied to clinical studies of chronic asthma.

## 4. Conclusions

The Notch signaling family is a conserved and essential signaling family in a plethora of biologic events. In allergic airway inflammation, Notch regulates the Th-skewed inflammatory outcome by coordinating messages originating from the inflammatory milieu. By Jagged and DLL engagement of the Notch receptors on T cells, Notch orchestrates the generation of Th2 versus Th1/17 and Tregs, which altogether elicit the allergic immune responses and regulate resolution of the inflammation in due course. Lack of DLL4-derived signaling leads to uncontrolled inflammation and aggravates pathology due to insufficient Treg differentiation and immune suppression.

On the other hand, in chronic allergic inflammation of the lung, Notch alleviates severity of the asthmatic phenotype by Treg-expressed DLL4 signaling. Repeated allergic inflammation in the lung causes airway remodeling, including inflammatory angiogenesis of the airways. Tregs-derived DLL4 signaling inhibits aberrant EC remodeling angiogenesis of the airways and improves lung function.

In recent years, commensal microbiota and their metabolic products have been shown to shape the development of mucosal immunity [130,131,132,133,134]. Among them, gut microbacteria produce short-chain fatty acids (SCFAs), acetate, propionate and butyrate, by metabolizing polysaccharides and dietary fibers that are otherwise indigestible. Notably, SCFAs induce de novo generation and accumulation of colonic Tregs [135,136,137]. High-fiber diet and SCFA supplementation have been shown to protect against allergic inflammation in the lung [138,139]. To this aspect, we found that SCFAs decreased DC expression of Notch ligands Jagged1 and DLL4, suggesting a regulatory role for SCFAs in the Notch signaling pathway (MT Huang et al., unpublished data). Future investigation will uncover whether and how Notch can be modulated by SCFAs or molecules to gain its beneficial effects in preventing allergic airway inflammation.

## Figures and Tables

**Table 1:** Harnessing Notch signaling in animal models of allergic airway inflammation.

Delta-Like Ligand (DLL)	Jagged	γ-Secretase Inhibitor (GSI)
DLL4 was up-regulated on BMDCs after RSV infection. Anti-DLL4 Ab-treated animals developed substantially increased AHR [68].	c-Kit-mutant mice had blunted Jagged2 expression on DCs and diminished Th2 & Th17 response to HDM allergic airway inflammation [62].	IL-4-deficient recipients of GSI-treated naive CD4^+^ T cells developed lower levels of AHR, reduced numbers of eosinophils, and lower Th2 cytokine [52].
Treatment of OVA-sensitized and challenged mice with Delta1-Fc decreased AHR and airway inflammation [53].	BMDCs pulsed with allergen upregulated the expression of Jagged1; transfer of these BMDCs induced AHR and eosinophilic airway inflammation. In vivo treatment with Jagged1-Fc enhanced AHR and airway inflammation [52].	GSI-treated CD8+ T cells failed to restore OVA-induced airway inflammation and AHR in OVA-sensitized recipient CD8−/− mice [53].
DLL1 ligation endowed antigen-presenting cell functions on mast cells, with expression of MHC-II and OX40L and ability to promote naive CD4^+^ T cell proliferation and differentiation into Th2 cells [80].	LPS-stimulated stromal cells induced upregulated expression of Jagged-1 by DCs, but not DLL4, which induced allergic airway inflammation [61].	Administration of GSI inhibited asthma phenotypes, including eosinophilic airway inflammation, goblet cell metaplasia, AHR, and serum IgE [51].
DCs upregulated DLL4 expression in response to cockroach allergen. Blocking DLL4 in vivo enhanced allergen-induced AHR, Th2 cytokine production and mucus secretion [67].	Jagged1 over-expression enhanced basal cell differentiation into secretory cells, but not ciliated cell differentiation [63].	GSI treatment suppressed Th17 responses and ameliorated the development of OVA-induced allergic asthma [77].
Mouse mast cells adhered to DLL1-expressing stromal cells, suggesting Notch ligands provided an adhesion function, facilitating mast cell recruitment into inflammatory sites [81].	Jagged1 inhibition mitigated Th2-dominated airway inflammation, whilst DLL4 blockage aggravated the asthma phenotypes due to impaired Treg induction. Adoptive transfer of DLL4-expressing antigen-presenting cells promoted Treg expansion [69].	
	Anti-DLL4 Ab-treated mice had decreased Th17 cells and mitigated airway inflammation [78].	

BMDCs: bone marrow-derived dendritic cells; DCs: dendritic cells; HDM: house dust mite; RSV: respiratory syncytial virus; AHR: airway hyperresponsiveness.
